# Supplementary material for: DLm6Am: A Deep-Learning-Based Tool for Identifying N6,2′-O-Dimethyladenosine Sites in RNA Sequences
Source: Int J Mol Sci. 2022 Sep 20;23(19):11026. doi: 10.3390/ijms231911026 (PMC9570463; doi:10.3390/ijms231911026)
Supplement: Supplementary file 1 [file ijms-23-11026-s001.zip › ijms-1881383-supplementary.pdf]

International Journal of
Molecular Sciences

MDPI

Supplementary Materials

DLm6Am: A Deep-Learning-Based Tool for Identifying N6,2′-O-Dimethyladenosine Sites in RNA Sequences

Zhengtao Luo [1,†], Wei Su [2,†], Liliang Lou [1], Wangren Qiu [1], Xuan Xiao [1,*] and Zhaochun Xu [1,*]

1 Computer Department, Jingdezhen Ceramic University, Jingdezhen 333403, China
2 School of Life Science and Technology and Center for Informational Biology, University of Electronic Science and Technology of China, Chengdu 610054, China
* Correspondence: xiaoxuan@jcu.edu.cn (X.X.); xuzhaochun@jcu.edu.cn (Z.X.)
† These authors contributed equally to this work.

Supplementary Note S1

Here, we plotted learning curves to infer the training set size that can make the model reach a stable state based on the cross-validation scores. In this plot, the abscissa axis represents the training set size, and the vertical axis represents the cross-validation scores. Specifically, we randomly sampled 500 positive samples and 500 negative samples as training set from the benchmark dataset, and the remaining samples as testing set. And then, the five-fold cross-validation was implemented 5 times on the current training set to obtain the average metric values, such as ACC and AUROC. Next, we randomly moved 100 positive samples and 100 negative samples from the testing set to training set, and implemented five-fold cross-validation 5 times again on training set, this process is repeated until the ratio of the training set and testing set is 9:1.

It can be seen from **Figure S1**, with the increase of the training set size, the ACC values show an increase trend under the overall. When the sample size was 2800, ACC reaches the maximum. Moreover, the AUROC values increase as the training set size increases, but run to steady when the training set size reaches 2800, i.e. the ratio of the number of training and testing set is close to 8:2, indicating that the ratio of 8:2 used for training/testing in this manuscript is reasonable. This ratio is widely used in construction of training/testing set for modification site identification, such as multi-type post-translational modification sites in rice (PMID: 34864888), RNA D sites (PMID: 31077296), and so on.

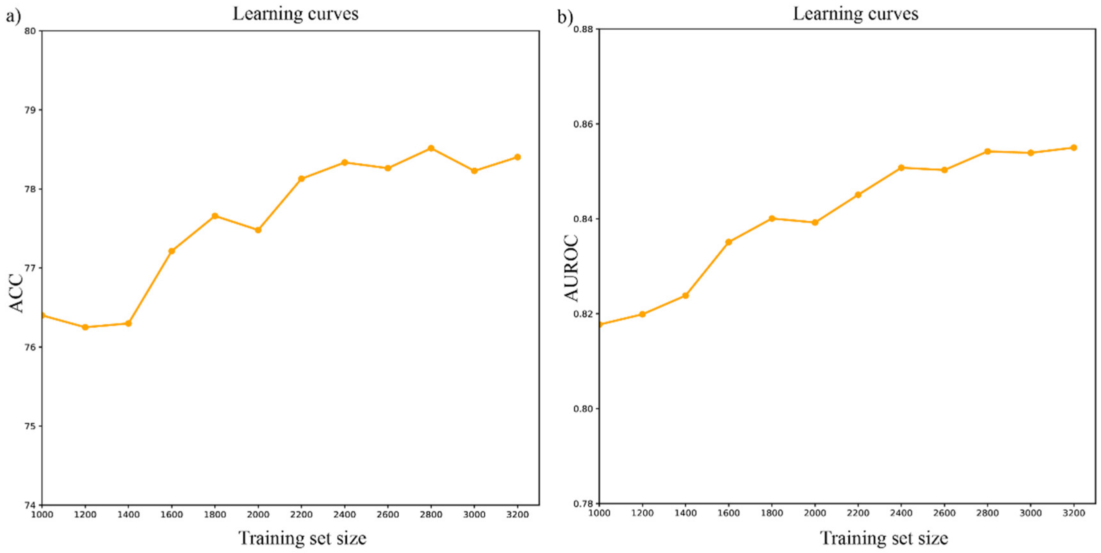

Figure S1. The learning curves plotted by the training set size and cross-validation scores.

Supplementary Note S2

Supplementary Note S2 provides more details regarding actual model configurations used, such as layer sizes, depth, number of parameters, etc.

1.RF
RandomForestClassifier:(n_estimators=500)

2.SVM
SVC:(C=1.2, probability=True)

3.XGBoost
GradientBoostingClassifier:(n_estimators=250)

4.CNN
Model: "sequential"

Layer (type)	Output Shape	Param #
conv1d (Conv1D)	(None, 38, 32)	1056
conv1d_1 (Conv1D)	(None, 35, 32)	4128
max_pooling1d (MaxPooling1D)	(None, 17, 32)	0
dropout (Dropout)	(None, 17, 32)	0
flatten (Flatten)	(None, 544)	0
dense (Dense)	(None, 10)	5450
dense_1 (Dense)	(None, 2)	22

Total params: 10,656
Trainable params: 10,656
Non-trainable params: 0

5.BiLSTM
Model: "sequential"

Layer (type)	Output Shape	Param #
bidirectional (Bidirectional	multiple	10496
bidirectional_1 (Bidirection	multiple	10368
flatten (Flatten)	multiple	0
dropout (Dropout)	multiple	0

```
dense (Dense)              multiple              13130
_________________________________________________________________
dense_1 (Dense)            multiple              22
=================================================================
Total params: 34,016
Trainable params: 34,016
Non-trainable params: 0
```

6.CNN-BiLSTM

```
net:((lstm): LSTM(8, 32, num_layers=2, batch_first=True, bidirectional=True)
(lstm_fc): Linear(in_features=2624, out_features=256, bias=True)
(block2): Sequential(
(0): Conv2d(1, 32, kernel_size=(4, 4), stride=(1, 1))
(1): Conv2d(32, 64, kernel_size=(4, 4), stride=(1, 1))
(2): MaxPool2d(kernel_size=2, stride=2, padding=0, dilation=1, ceil_mode=False)
)
(block3): Sequential(
(0): Dropout(p=0.5, inplace=False)
(1): Linear(in_features=1344, out_features=128, bias=True)
(2): LeakyReLU(negative_slope=0.01)
(3): Linear(in_features=128, out_features=12, bias=True)
(4): LeakyReLU(negative_slope=0.01)
(5): Linear(in_features=12, out_features=2, bias=True)
(6): Softmax(dim=1)
))
```

7.CNN-BiLSTM-attention

```
net:((encoder_layer): TransformerEncoderLayer(
(self_attn): MultiheadAttention(
  (out_proj): NonDynamicallyQuantizableLinear(in_features=8, out_features=8, bias=True)
)
(linear1): Linear(in_features=8, out_features=2048, bias=True)
(dropout): Dropout(p=0.1, inplace=False)
(linear2): Linear(in_features=2048, out_features=8, bias=True)
(norm1): LayerNorm((8,), eps=1e-05, elementwise_affine=True)
(norm2): LayerNorm((8,), eps=1e-05, elementwise_affine=True)
(dropout1): Dropout(p=0.1, inplace=False)
(dropout2): Dropout(p=0.1, inplace=False)
)
(transformer_encoder): TransformerEncoder(
(layers): ModuleList(
  (0): TransformerEncoderLayer(
      (self_attn): MultiheadAttention(
        (out_proj): NonDynamicallyQuantizableLinear(in_features=8, out_features=8, bias=True)
      )
      (linear1): Linear(in_features=8, out_features=2048, bias=True)
      (dropout): Dropout(p=0.1, inplace=False)
      (linear2): Linear(in_features=2048, out_features=8, bias=True)
      (norm1): LayerNorm((8,), eps=1e-05, elementwise_affine=True)
      (norm2): LayerNorm((8,), eps=1e-05, elementwise_affine=True)
      (dropout1): Dropout(p=0.1, inplace=False)
      (dropout2): Dropout(p=0.1, inplace=False)
```

```
  )
)
)
(lstm): LSTM(8, 32, num_layers=2, batch_first=True, bidirectional=True)
(lstm_fc): Linear(in_features=2624, out_features=256, bias=True)
(block2): Sequential(
(0): Conv2d(1, 32, kernel_size=(4, 4), stride=(1, 1))
(1): Conv2d(32, 64, kernel_size=(4, 4), stride=(1, 1))
(2): MaxPool2d(kernel_size=2, stride=2, padding=0, dilation=1, ceil_mode=False)
)
(block3): Sequential(
(0): Dropout(p=0.5, inplace=False)
(1): Linear(in_features=1344, out_features=128, bias=True)
(2): LeakyReLU(negative_slope=0.01)
(3): Linear(in_features=128, out_features=12, bias=True)
(4): LeakyReLU(negative_slope=0.01)
(5): Linear(in_features=12, out_features=2, bias=True)
(6): Softmax(dim=1)
))
```

8.DLm6Am

```
net1:((encoder_layer): TransformerEncoderLayer(
(self_attn): MultiheadAttention(
  (out_proj): NonDynamicallyQuantizableLinear(in_features=8, out_features=8, bias=True)
)
(linear1): Linear(in_features=8, out_features=2048, bias=True)
(dropout): Dropout(p=0.1, inplace=False)
(linear2): Linear(in_features=2048, out_features=8, bias=True)
(norm1): LayerNorm((8,), eps=1e-05, elementwise_affine=True)
(norm2): LayerNorm((8,), eps=1e-05, elementwise_affine=True)
(dropout1): Dropout(p=0.1, inplace=False)
(dropout2): Dropout(p=0.1, inplace=False)
)
(transformer_encoder): TransformerEncoder(
(layers): ModuleList(
  (0): TransformerEncoderLayer(
      (self_attn): MultiheadAttention(
        (out_proj): NonDynamicallyQuantizableLinear(in_features=8, out_features=8, bias=True)
      )
      (linear1): Linear(in_features=8, out_features=2048, bias=True)
      (dropout): Dropout(p=0.1, inplace=False)
      (linear2): Linear(in_features=2048, out_features=8, bias=True)
      (norm1): LayerNorm((8,), eps=1e-05, elementwise_affine=True)
      (norm2): LayerNorm((8,), eps=1e-05, elementwise_affine=True)
      (dropout1): Dropout(p=0.1, inplace=False)
      (dropout2): Dropout(p=0.1, inplace=False)
  )
)
)
(lstm): LSTM(8, 32, num_layers=2, batch_first=True, bidirectional=True)
(lstm_fc): Linear(in_features=2624, out_features=256, bias=True)
(block2): Sequential(
(0): Conv2d(1, 32, kernel_size=(4, 4), stride=(1, 1))
```

```
(1): Conv2d(32, 64, kernel_size=(4, 4), stride=(1, 1))
(2): MaxPool2d(kernel_size=2, stride=2, padding=0, dilation=1, ceil_mode=False)
)
(block3): Sequential(
(0): Dropout(p=0.5, inplace=False)
(1): Linear(in_features=1344, out_features=128, bias=True)
(2): LeakyReLU(negative_slope=0.01)
(3): Linear(in_features=128, out_features=12, bias=True)
(4): LeakyReLU(negative_slope=0.01)
(5): Linear(in_features=12, out_features=2, bias=True)
(6): Softmax(dim=1)
))

net2:((encoder_layer): TransformerEncoderLayer(
(self_attn): MultiheadAttention(
  (out_proj): NonDynamicallyQuantizableLinear(in_features=8, out_features=8, bias=True)
)
(linear1): Linear(in_features=8, out_features=2048, bias=True)
(dropout): Dropout(p=0.1, inplace=False)
(linear2): Linear(in_features=2048, out_features=8, bias=True)
(norm1): LayerNorm((8,), eps=1e-05, elementwise_affine=True)
(norm2): LayerNorm((8,), eps=1e-05, elementwise_affine=True)
(dropout1): Dropout(p=0.1, inplace=False)
(dropout2): Dropout(p=0.1, inplace=False)
)
(transformer_encoder): TransformerEncoder(
(layers): ModuleList(
  (0): TransformerEncoderLayer(
      (self_attn): MultiheadAttention(
        (out_proj): NonDynamicallyQuantizableLinear(in_features=8, out_features=8, bias=True)
      )
      (linear1): Linear(in_features=8, out_features=2048, bias=True)
      (dropout): Dropout(p=0.1, inplace=False)
      (linear2): Linear(in_features=2048, out_features=8, bias=True)
      (norm1): LayerNorm((8,), eps=1e-05, elementwise_affine=True)
      (norm2): LayerNorm((8,), eps=1e-05, elementwise_affine=True)
      (dropout1): Dropout(p=0.1, inplace=False)
      (dropout2): Dropout(p=0.1, inplace=False)
  )
)
)
(lstm): LSTM(8, 32, num_layers=2, batch_first=True, bidirectional=True)
(lstm_fc): Linear(in_features=2624, out_features=256, bias=True)
(block2): Sequential(
(0): Conv2d(1, 32, kernel_size=(4, 4), stride=(1, 1))
(1): Conv2d(32, 64, kernel_size=(4, 4), stride=(1, 1))
(2): MaxPool2d(kernel_size=2, stride=2, padding=0, dilation=1, ceil_mode=False)
)
(block3): Sequential(
(0): Dropout(p=0.5, inplace=False)
(1): Linear(in_features=1344, out_features=128, bias=True)
(2): LeakyReLU(negative_slope=0.01)
(3): Linear(in_features=128, out_features=12, bias=True)
(4): LeakyReLU(negative_slope=0.01)
```

```
(5): Linear(in_features=12, out_features=2, bias=True)
(6): Softmax(dim=1)
))

net3:((encoder_layer): TransformerEncoderLayer(
(self_attn): MultiheadAttention(
  (out_proj): NonDynamicallyQuantizableLinear(in_features=8, out_features=8, bias=True)
)
(linear1): Linear(in_features=8, out_features=2048, bias=True)
(dropout): Dropout(p=0.1, inplace=False)
(linear2): Linear(in_features=2048, out_features=8, bias=True)
(norm1): LayerNorm((8,), eps=1e-05, elementwise_affine=True)
(norm2): LayerNorm((8,), eps=1e-05, elementwise_affine=True)
(dropout1): Dropout(p=0.1, inplace=False)
(dropout2): Dropout(p=0.1, inplace=False)
)
(transformer_encoder): TransformerEncoder(
(layers): ModuleList(
  (0): TransformerEncoderLayer(
      (self_attn): MultiheadAttention(
        (out_proj): NonDynamicallyQuantizableLinear(in_features=8, out_features=8, bias=True)
      )
      (linear1): Linear(in_features=8, out_features=2048, bias=True)
      (dropout): Dropout(p=0.1, inplace=False)
      (linear2): Linear(in_features=2048, out_features=8, bias=True)
      (norm1): LayerNorm((8,), eps=1e-05, elementwise_affine=True)
      (norm2): LayerNorm((8,), eps=1e-05, elementwise_affine=True)
      (dropout1): Dropout(p=0.1, inplace=False)
      (dropout2): Dropout(p=0.1, inplace=False)
  )
)
)
(lstm): LSTM(8, 32, num_layers=2, batch_first=True, bidirectional=True)
(lstm_fc): Linear(in_features=2624, out_features=256, bias=True)
(block2): Sequential(
(0): Conv2d(1, 32, kernel_size=(4, 4), stride=(1, 1))
(1): Conv2d(32, 128, kernel_size=(4, 4), stride=(1, 1))
(2): MaxPool2d(kernel_size=2, stride=2, padding=0, dilation=1, ceil_mode=False)
)
(block3): Sequential(
(0): Dropout(p=0.5, inplace=False)
(1): Linear(in_features=2432, out_features=256, bias=True)
(2): LeakyReLU(negative_slope=0.01)
(3): Linear(in_features=256, out_features=24, bias=True)
(4): LeakyReLU(negative_slope=0.01)
(5): Linear(in_features=24, out_features=2, bias=True)
(6): Softmax(dim=1)
))
```

Supplementary Note S3

The additional performance gains of the DLm6Am model may be achieved while varying model complexity (e.g., number of layers/nodes), as well varying/increasing training set size. As seen in **Supplementary Note S1**, we inferred the optimal ratio (0.789, close to 8:2) of the training/testing set through learning curves. Therefore, here, we only analyzed the effect of varying model complexity (e.g. the number of layers and nodes for BiLSTM as well the number of layers and filters for CNN) on performance under this ratio of the training/testing set. The corresponding results were listed in **Table S1**. It can be seen that DLm6Am could achieve better prediction performance under the model configurations used in the manuscript, i.e. the number of layers for BiLSTM was set as 2, the number of nodes for BiLSTM as 32, the number of layers for CNN as 2 and the number of filters for CNN as 32.

Table S1. Model performance when varying model complexity.

BiLSTM		CNN		Metrics					
No.of layers	No.of nodes	No. of layers	No. of filters	Acc(%)	Sn(%)	Sp(%)	MCC	AUC	PR
2	16	2	16	78.44	79.49	77.38	0.5688	0.8493	0.8523
2	16	2	32	78.61	78.01	79.21	0.5723	0.8525	0.8524
2	16	2	64	78.37	78.79	77.94	0.5673	0.85	0.8503
2	32	2	16	78.68	79.28	78.08	0.5737	0.8504	0.8511
**2**	**32**	**2**	**32**	**79.07**	**79.21**	**78.93**	**0.5814**	**0.8545**	**0.8532**
2	32	2	64	78.79	79.7	77.87	0.5759	0.852	0.8535
2	64	2	16	78.05	79.63	76.46	0.5612	0.8446	0.8449
2	64	2	32	78.37	77.73	79	0.5673	0.8495	0.8507
2	64	2	64	78.54	79.07	78.01	0.5709	0.851	0.8519
2	16	3	16	78.22	77.66	78.79	0.5645	0.8495	0.8517
2	16	3	32	78.68	78.01	79.35	0.5737	0.8531	0.8544
2	16	3	64	78.05	76.96	79.14	0.5611	0.8437	0.8442
2	32	3	16	77.94	79.77	76.11	0.5592	0.8471	0.8446
2	32	3	32	78.4	78.79	78.01	0.568	0.8468	0.8454
2	32	3	64	78.19	78.01	78.37	0.5638	0.8465	0.8485
2	64	3	16	77.52	77.31	77.73	0.5504	0.8424	0.8383
2	64	3	32	78.4	78.15	78.65	0.568	0.8484	0.847
2	64	3	64	78.26	78.93	77.59	0.5652	0.8481	0.847
3	16	2	16	78.19	75.97	80.41	0.5643	0.8495	0.8496
3	16	2	32	78.72	79.07	78.37	0.5744	0.8512	0.8548
3	16	2	64	77.98	76.96	79	0.5597	0.8484	0.8505
3	32	2	16	78.47	78.79	78.15	0.5694	0.8483	0.8512
3	32	2	32	78.37	78.93	77.8	0.5673	0.8485	0.8495
3	32	2	64	78.26	78.58	77.94	0.5652	0.8493	0.852
3	64	2	16	78.33	79	77.66	0.5666	0.8463	0.8453
3	64	2	32	78.75	79.56	77.94	0.5751	0.8522	0.8516
3	64	2	64	78.15	78.79	77.52	0.5631	0.8496	0.8496
3	16	3	16	78.29	79	77.59	0.5659	0.8525	0.8525
3	16	3	32	77.87	80.2	75.55	0.558	0.8474	0.849
3	16	3	64	78.12	78.86	77.38	0.5624	0.8452	0.8451
3	32	3	16	78.26	80.27	76.25	0.5656	0.8521	0.8525
3	32	3	32	78.51	78.29	78.72	0.5701	0.8492	0.8499
3	32	3	64	78.15	79.14	77.17	0.5632	0.8501	0.8526
3	64	3	16	77.87	78.22	77.52	0.5574	0.8447	0.8436
3	64	3	32	78.4	77.8	79	0.568	0.8465	0.8457
3	64	3	64	78.15	78.37	77.94	0.5631	0.8462	0.8425

Supplementary Note S4

We compared the prediction performance of between DLm6Am and other combinations of different base classifiers such as SVM, RF, XGBoost, LSTM, and CNN. The ensemble classification results are listed in the **Table S2**. It can be seen that the DLm6Am achieved better prediction performance than other ensemble combinations.

Table S2. Model performance when varying model complexity.

Model	Acc(%)	Sn(%)	Sp(%)	MCC	AUROC	AUPR
RF-SVM-XGBoost	75.86	76.46	75.26	0.5173	0.8314	0.8287
RF-SVM-BiLSTM	75.09	75.76	74.42	0.5018	0.8319	0.8266
RF-SVM-CNN	76.43	77.17	75.69	0.5286	0.8508	0.8505
RF-SVM-CNN+BiLSTM	75.58	75.83	75.33	0.5116	0.8496	0.8473
RF-XGBoost-BiLSTM	76.39	76.74	76.04	0.5278	0.8331	0.8303
RF-XGBoost-CNN	76.64	75.19	78.08	0.533	0.8441	0.8469
RF-XGBoost-CNN+BiLSTM	76.32	74.77	77.87	0.5267	0.8443	0.8446
RF-BiLSTM-CNN	77.17	77.45	76.89	0.5433	0.8456	0.8449
RF-BiLSTM-CNN+BiLSTM	76.29	76.67	75.9	0.5257	0.8447	0.8419
RF-CNN-CNN+BiLSTM	76.29	75.41	77.17	0.5258	0.8534	0.8455
SVM-XGBoost-BiLSTM	75.58	76.46	74.7	0.5117	0.8335	0.8287
SVM-XGBoost-CNN	76.85	78.37	75.33	0.5372	0.8448	0.8472
SVM-XGBoost-CNN+BiLSTM	76.57	77.45	75.69	0.5314	0.8448	0.845
SVM-BiLSTM-CNN	76.36	76.81	75.9	0.5272	0.847	0.8455
SVM-BiLSTM-CNN+BiLSTM	76.43	76.89	75.97	0.5286	0.8446	0.8413
SVM-CNN-CNN+BiLSTM	76.5	76.96	76.04	0.53	0.8513	0.8504
XGBoost-BiLSTM-CNN	76.6	77.66	75.55	0.5322	0.8445	0.8449
XGBoost-BiLSTM-CNN+BiLSTM	76.07	76.6	75.55	0.5215	0.8451	0.8428
XGBoost-CNN-CNN+BiLSTM	77.1	77.31	76.89	0.5419	0.8522	0.8466
BiLSTM-CNN-CNN+BiLSTM	76.74	79.07	74.42	0.5355	0.8502	0.8486
**DLm6Am**	**79.07**	**79.21**	**78.93**	**0.5814**	**0.8545**	**0.8532**
